# On the Physiology and Diseases of the Pancreas

**Published:** 1854-06

**Authors:** M. Bernard


### *On the Physiology and Disease of the Pancreas.*

BY DR. BRIGHT AND M. BERNARD.

[In 1832, three papers were read successively before the Medico-Chirurgical Society on this obscure but interesting subject; the authors being Dr. Bright, Mr. Lloyd, and Dr. Elliotson. These gentlemen directed attention to a particular and rare symptom.]

"The symptoms to which I refer," said Dr. Bright, who was the author of the first communication, "is a peculair condition of the alvine evacuation; a portion more or less considerable assuming the character of an oily substance resembling fat, which either passes separately from the bowels, or soon divides itself from the general mass, and lies upon the surface, sometimes forming a thick crust, particularly about the edges of the vessel, if the fæces are of a semi-fluid consistence; sometimes floating like globules of tallow which have melted and become cold; and sometimes assuming the form of a thin fatty pellicle over the whole, or over the fluid parts in which the more solid figured fæces are deposited. This oily matter has generally a slight yellow tinge, and a most digustingly fœtid odor."

The importance of Dr. Bright's views, as bearing upon recent investigations, will be seen in the following summary of them. He says:—

"When we draw a comparison between the three foregoing cases, a very close analogy, or even identity, in many circumstances, may be traced. In all of them chronic ailment terminated, sooner or later, in jaundice; and in all of them a great peculiarity in the character of the dejections existed. In the result of the examination after death, we have likewise some circumstances which coincide in all—obstructed biliary ducts; the liver gorged with bile; fungoid disease attacking the head of the pancreas; and malignant ulceration on the surface of the duodenum. The question to be solved is, upon which of the conditions indicated or caused by these morbid changes, if upon either, the peculiarity of the evacuations depended? That the obstruction of the biliary ducts, or even the total absence of all indication of biliary secretion, is not usually attended by the same peculiarity in the evacuations, many cases which have been cautiously detailed by various authors, and many which we have all observed, bear sufficient testimony; and I was therefore induced to ascribe it either to the existence of malignant disease, or to that disease being situated in the pancreas. That the simple fact of malignant disease existing, is not necessarily productive of such appearances in the feculent matter, I infer from cases both of that form of disease and of melanosis in the liver to a very great extent being, within the scope of my experience, unaccompanied by any such discharge, though the evacuations were submitted to the most rigid observation. That simple ulceration in the bowels, to any known extent, is not attended by any such symptom, I am led to believe, from knowing that neither in the most extensive ulceration of the large intestines in cases of dysentery, nor in the worst cases of ulceration of the small intestines in fever, in diarrhœa, or in phthisis, does anything of the kind usually occur.—Whether, however, malignant ulceration of the mucous membrane is accompanied by this symptom, I cannot assert, though I have often seen most extensive ulcers of the pylorus and of the rectum, where, although the evacuations were attentively observed, such fatty matter was not detected. As, however, a malignant ulceration of the membrane did exist in each of the foregoing cases, it is not impossible that this was the cause of this symptom; but we must bear in mind that such ulcerations are by no means uncommon, and that the phenomenon of which I am speaking is uncommon; and that in each of the cases it was accompanied by another morbid appearance, which is not common—namely, the malignant disease of the pancreas. The fact of the intestinal ulceration having, in each case, occupied the duodenum, does, however, somewhat diminish the weight of this observation, for that certainly is not so frequent occurrence."—*Brit. & For. Medico-Chir. Review, July*, 1853, *p.* 154.

